# Adsorption Properties of Granular Activated Carbon-Supported Titanium Dioxide Particles for Dyes and Copper Ions

**DOI:** 10.1038/s41598-018-24891-1

**Published:** 2018-04-24

**Authors:** Xin Zheng, Nannan Yu, Xiaopeng Wang, Yuhong Wang, Linshan Wang, Xiaowu Li, Xiaomin Hu

**Affiliations:** 1 College of Science, Northeastern University, Shenyang, 110819 China; 2 School of Materials Science and Engineering, Northeastern University, Shenyang, 110819 China; 3 Shenyang Institute of Special Equipment Inspection and Research, Shenyang, 110035 China; 4 Hunnan Branch, Shenyang Environmental Protection Bureau, Shenyang, 110015 China; 5 School of Resources and Civil Engineering, Northeastern University, Shenyang, 110819 China

## Abstract

In the present paper, granular activated carbon (GAC) supported titanium dioxide (TiO_2_@GAC) particles were prepared by sol-gel process. Their performance in simultaneous adsorption of dye and Cu^2+^ from wastewater was studied. X-ray diffraction (XRD) indicated that TiO_2_ of the TiO_2_@GAC microsphere is anatase type, and Fourier transform infrared spectroscopy (FT-IR) showed that the samples have obvious characteristic peaks in 400–800 cm^−1^, which indicated that there are Ti-O-Ti bonds. The experimental results showed that the adsorption of TiO_2_@GAC for Methylene blue (MB) and Cu^2+^ were favorable under acidity condition, the adsorption of Methyl orange (MO) was favorable under alkalecent condition. The reaction kinetics of TiO_2_@GAC for MO, MB and Cu^2+^ were well described as pseudo-second-order kinetic model; The reaction isotherms for MO, MB and Cu^2+^ were well fitted by Langmuir model. The maximum adsorption capacity of TiO_2_@GAC for MO, MB and Cu^2+^ in the single systems were 32.36 mg/g, 25.32 mg/g and 23.42 mg/g, respectively. As for adsorption, Cu^2+^ had a suppression effect on MB, and a promotion effect on MO, however, the impact of MO and MB on Cu^2+^ were negligible.

## Introduction

With the rapid development of industry, there is more and more concern about toxic dyes and heavy metal ions in untreated waste water from industrial production processes^1^. Most dyes and their intermediates have teratogenic, carcinogenic or mutagenic effects and high biological toxicity. Some dyes even become chemicals for carcinogenicity tests. Meanwhile, due to their wide application, the released dyes and dye intermediates have caused serious damages to the external environment, which are very difficult to control^2^. As one of the most common heavy metal ions, too much Cu^2+^ in the human body will cause gastrointestinal problems, liver and kidney damage, nausea, hair loss, severe headache and even death^3^. Therefore, how to remove organic dyes and heavy metal ions in wastewater has become a hot topic in environmental protection. There are many removal methods, such as adsorption method^4^, ion exchange method^5^ and chemical precipitation method^6^. Among these methods, the adsorption method is widely used because of its high adsorption efficiency, simple operation and recoverability^7^.

In view of the adsorption method, scholars have studied the adsorption performance of various adsorbent materials for contaminants. Tang *et al*.^4^ studied the simultaneous adsorption of atrazine and Cu^2+^ by magnetic carbon nanotubes. Asuha *et al*.^7^ investigated the adsorption performance of TiO_2_ for methyl orange and Cr(VI). Among these adsorbent materials, titanium dioxide is very promising for environment-purifying applications since ion doping and immobilization^8–11^. However, titanium dioxide is present in the form of powder and is difficult to be separated from aqueous solution for recovery and reuse. Due to its high mechanical strength, wide pore size distribution and high adsorption capacity, granular activated carbon can be effectively used as a carrier of TiO_2_. The combination of granular activated carbon and titanium dioxide can accelerate the settling rate and enhance the adsorption capacity, making up for the shortcomings of TiO_2_ and thereby allowing wide application in wastewater treatment^12^. Most of the previous literature focused on the removal of contaminants with this material in a single system^12–14^. However, the application of this material in more complex multivariate systems is rarely reported. In this paper, Cu^2+^, MO and methylene blue (MB) were selected to create a mixed system of heavy metals and dyes. The adsorption capacity of TiO_2_@GAC for Cu^2+^ and dyes was investigated. The adsorption performance of TiO_2_@GAC under the influence of pH, initial concentration of dyes/Cu^2+^ and time was studied in detail.

## Experimental Sections

### Materials and Instruments

Ethyl titanate, granular activated carbon, anhydrous ethanol, acetic acid, hydrochloric acid, methyl orange and metallic copper (Sinopharm Chemical Reagent Co., Ltd., analytical reagent); methylene blue (Guangdong Xilong Scientific Co., Ltd., analytical reagent). Secondary deionized water was used for all experiments.

Fourier transform infrared spectroscopy (Bruker, Germany); XRD-6000 diffractometer (Cu^2+^ Kα radiation, λ = 0.15406 nm, PANalytical, Holland); scanning electron microscope (SSX-550, Shimadzu Corporation); UV visible spectrophotometer (model 712, Shanghai Third Analytical Instrument Factory); atomic absorption spectrometer (TAS-990, Beijing Persee General Instrument Co., Ltd.).

### Pretreatment of granular activated carbon

The granular activated carbon (with an average particle size of 3 mm) was first washed with deionized water until the washings were colorless, so as to remove the ash. Then it was soaked in nitric acid for 24 h to remove organic matter and other impurities. Finally, it was washed with deionized water until the pH was neutral, and dried in a vacuum oven at 80 °C.

### Preparation of supported titanium dioxide

18 mL of titanium tetrabutyl titanate, 45 mL of anhydrous ethanol and 3 mL of acetic acid were mixed to prepare solution A; 45 mL of anhydrous ethanol and 8 mL of deionized water (adjusted to pH 2–3 with 0.1 mol nitric acid) was mixed to prepare solution B. 5 g of granular activated carbon was weighed and added to solution A. Solution B was slowly added to solution A with a separatory funnel under vigorous stirring. After the addition of solution B, the mixture was stirred to form a sol and was then allowed to stand for 2 days to form a jelly-like gel. The gel was dried in a vacuum oven at 90 °C, calcined at 250 °C for 1 h in an air atmosphere and then calcined at 600 °C for 2 h in a nitrogen atmosphere in a tube furnace to obtain TiO_2_@GAC.

### Analysis of TiO_2_ on TiO_2_@GAC

The method for analyzing TiO_2_ on GAC was described in detail by El-Sheikh *et al*.^15^. 0.1 g dried TiO_2_@GAC sample were weighed (±0.1 mg) in a Teflon tube, and 3.0 ml 18.0 M H_2_SO_4_, 0.04 g CuSO_4_ and 0.35 g K_2_SO_4_ were added to the tube. The Teflon tube with sample was digested in a microwave oven for 5 min. Then the tube was added in 7 ml water and centrifuged at 3000 rpm to remove residual carbon. The supernatant was mixed with 1.00 ml 30% H_2_O_2_, and diluted with water to 10.00 ml. Absorbance of the solution was detected at 410 nm.

### Adsorption experiment

10 mL of a single or binary solution with a certain concentration was added to a centrifuge tube. After the addition of 10 mg of TiO_2_@GAC particles, the tube was centrifuged. The supernatant was then removed and the concentration was measured. In the experiment, the absorbance of MO and MB was measured with a UV-Vis spectrophotometer (the maximum absorption wavelength of MO was 464 nm and the MB was 664 nm). The concentration of Cu^2+^ was measured with an atomic absorption spectrophotometer. The adsorption rate was calculated with the following formula:

1 $$\eta =\frac{{C}_{0}-{C}_{t}}{{C}_{0}}\times 100 \% $$

where C_0_ represents the concentration before adsorption and C_t_ represents the concentration after adsorption.

## Results and Discussion

**Figure 1:**
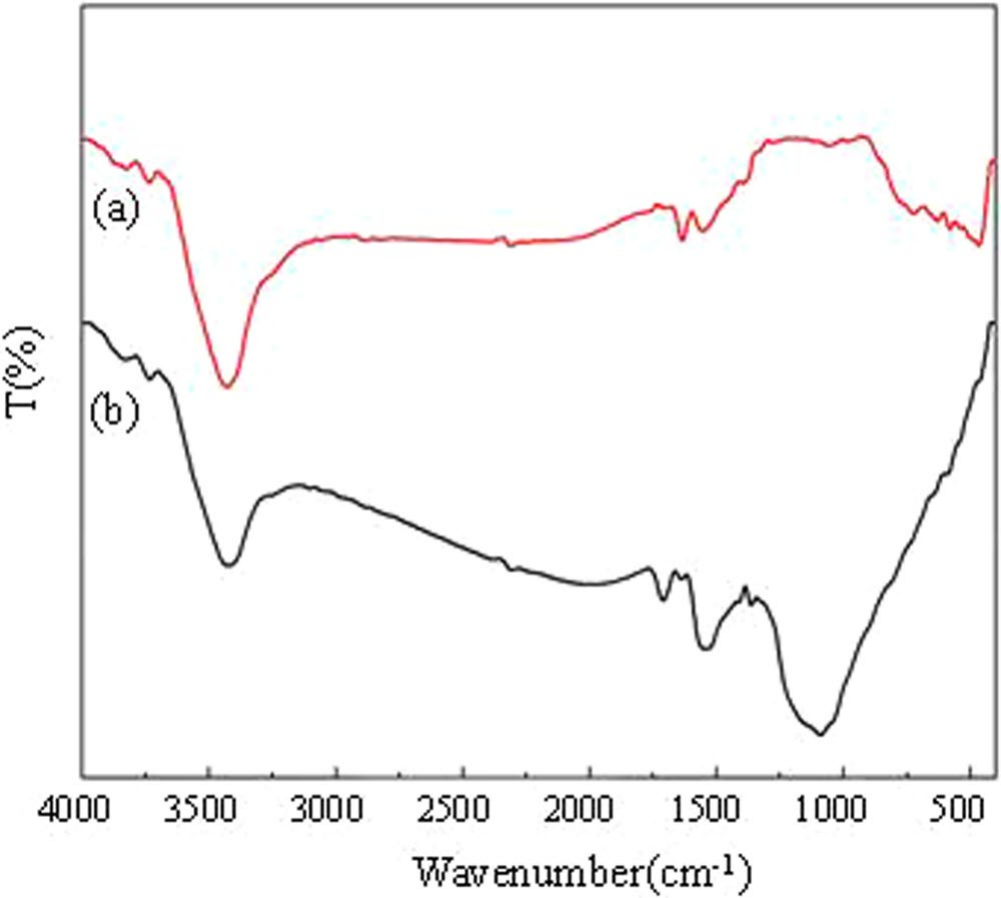
FT-IR spectra of TiO_2_@GAC (**a**) and GAC (**b**).

Figure 1 shows the infrared absorption spectra (FT-IR) of TiO_2_@GAC and GAC. It can be observed from Fig. (1b) that GAC has four main absorption bands in the wavelength range of 4000–400 cm^−1^. The absorption peaks at 3400 cm^−1^ and 1600 cm^−1^are due to the O-H stretching vibrations. The absorption peak at 1726 cm^−1^ is due to the C=O stretching vibration, while the absorption peak at 1060 cm^−1^ is due to skeletal stretching vibrations^16^. In Fig. (1a), the absorption bands of TiO_2_@GAC in the range of 400–800 cm^−1^ were different from those of GAC. This is caused by the Ti-O stretching vibrations. The absorption band at 1060 cm^−1^ disappears because GAC was covered by TiO_2_. The FT-IR analysis gives preliminary evidence that titanium dioxide has been loaded on granular activated carbon.

**Figure 2:**
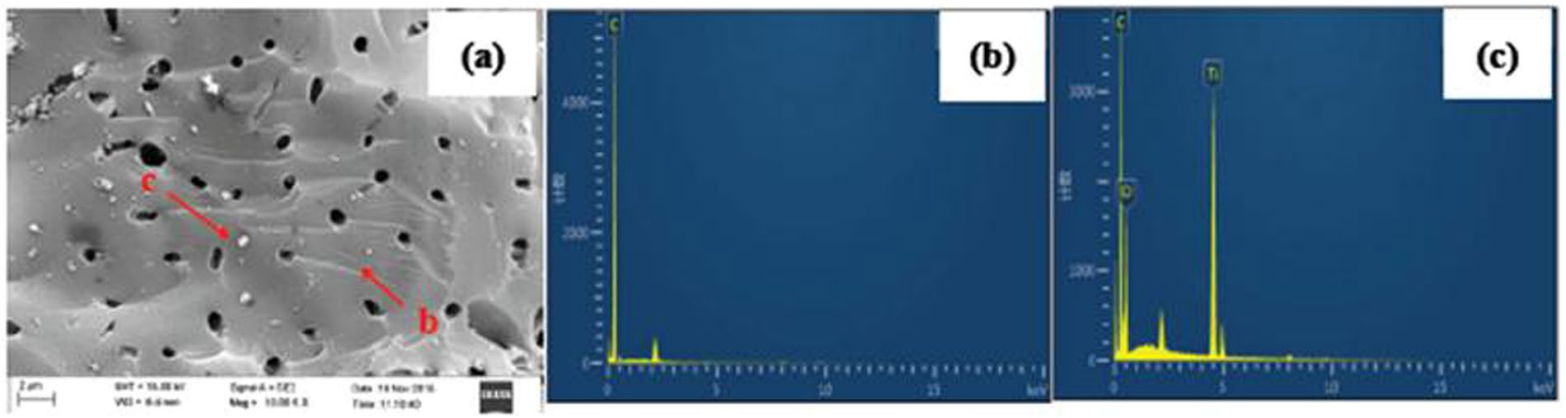
SEM (**a**) and EDS (**b** and **c**) images of TiO_2_@GAC.

Figure 2 shows the SEM and EDS images of the prepared supported titanium dioxide. It can be seen from Fig. 2(a) that the surface of GAC features a mesoporous structure and has been loaded with TiO_2_. It can be known from Fig. 2(b) that the EDS spectrum only contains the element C. Figure 2(c) suggests that three elements (Ti, C and O) are present in the EDS spectrum. Therefore, it can be concluded that TiO_2_ has been successfully loaded on GAC. The TiO_2_ contents on synthetic TiO_2_@GAC were in the range from 43.4 mg/g to 45.1 mg/g TiO_2_@GAC.

**Figure 3:**
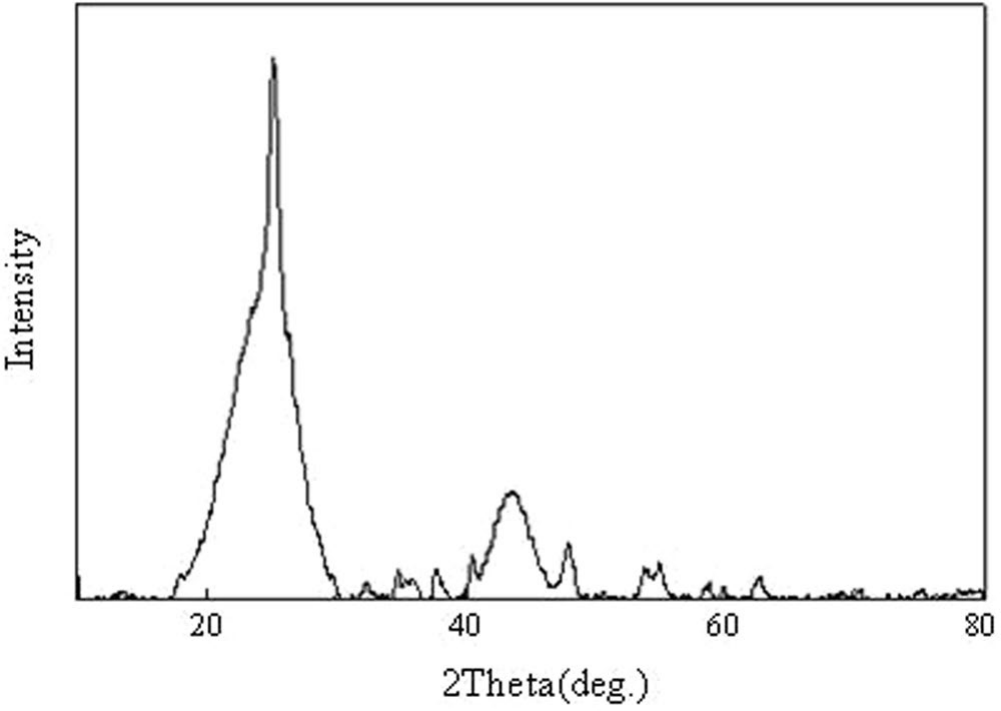
XRD patterns of TiO_2_@GAC.

Figure 3 shows the XRD analysis of TiO_2_@GAC. As shown in Fig. 3, in the 2θ range of 10° to 80°, there are six characteristic peaks of TiO_2_, which are 25.2°, 37.6°, 47.8°, 53.8°, 54.9° and 62.7°, respectively. According to JCDPS Card #16-629, they are the characteristic diffraction peaks of (101), (004), (200) (105), (211) and (204) planes of anatase TiO_2_, respectively^17^. There is a significant peak at 43.5°, which is a characteristic peak of activated carbon. This is probably because part of activated carbon has not been fully loaded. However, the above analysis already shows that TiO_2_ has been loaded onto GAC.

### Effect of pH

**Figure 4:**
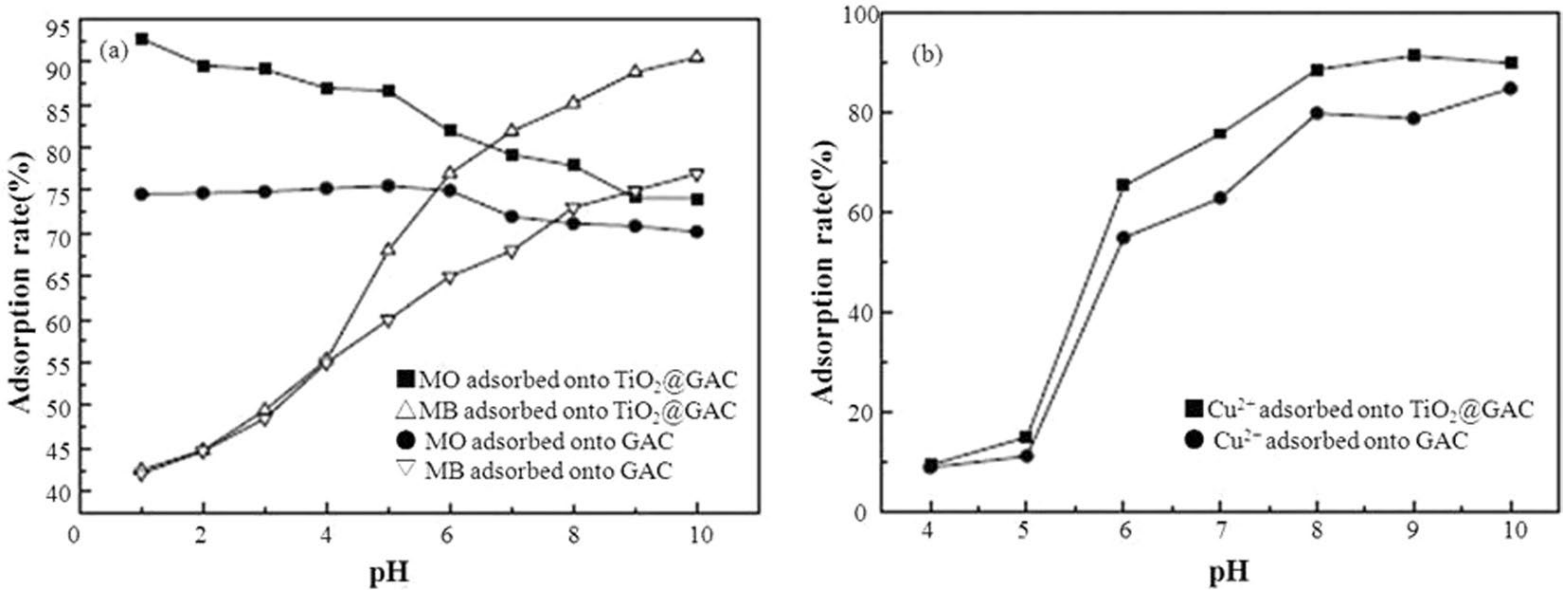
Effect of pH on the adsorption of MO, MB (**a**) and Cu^2+^ on TiO_2_@GAC and GAC (**b**).

Figure 4 shows the effect of pH on the adsorption performance of TiO_2_@GAC and GAC. It can be seen from Fig. 4 that, with constant changes in pH, the adsorption rate of TiO_2_@GAC for dyes and Cu^2+^ is higher than that of GAC. It can be concluded that the adsorption performance of TiO_2_@GAC for dyes and Cu^2+^ is better than that of GAC. It can be seen from Fig. 4(a) that, with the decrease of pH, the adsorption of MO on TiO_2_@GAC is not conducive to the adsorption of MB. With the increase of pH (1–10), the adsorption rate of MO decreases from 95.55% to 48.13%, while the adsorption rate of MB increases from 42.50% to 90.54%. These results can be explained by the theory of isoelectric point (pH_pzc_). According to literature, the pH_pzc_ of TiO_2_@GAC is about 6.0^18^.

When the pH value of the solution is less than the pH_pzc_, the surface of TiO_2_@GAC is positively charged (TiOH^2+^). On the contrary, when the pH value of the solution is greater than the pH_pzc_, the surface of TiO_2_@GAC is negatively charged (TiO^−^). Under acidic conditions, the MO molecule features a quinone structure with its sulfonate terminal negatively charged, facilitating its adsorption on the negatively charged surface of TiO_2_@GAC; while under alkaline conditions, the MO is negatively charged, resulting in an electrostatic repulsion toward the negatively charged TiO_2_@GAC, which hinders the adsorption of MO. In the case of MB, its molecular structure is positively charged. Under alkaline conditions, the negatively charged TiO_2_@GAC strongly adsorbed the positively charged MB, accelerating the removal of MB; while under acidic conditions, there is an electrostatic repulsion between the positive charges on the surface of TiO_2_@GAC and the positive charges on the MB molecule, which becomes one of the causes to the decreased removal efficiency. In addition, the decrease in the removal efficiency of MB under acidic conditions may also be due to the competition between H^+^ and MB on TiO_2_@GAC^19^.

It can be seen from Fig. 4 (b) that the adsorption rate of TiO_2_@GAC for Cu^2+^ increases with the increase of pH. The adsorption of Cu^2+^ can also be explained by pH_pzc_. As the pH increases, the Zeta potential of TiO_2_@GAC decreases. Because of the electrostatic attraction, the negatively charged TiO_2_@GAC (TiO^−^) (pH > 6) is conducive to the adsorption of Cu^2+^. Similarly, the positively charged TiO_2_@GAC (TiOH_2_^+^) (pH < 6) is not conducive to the adsorption of Cu^2+^. Furthermore, coprecipitation of Cu^2+^ occurs when the pH is higher than 6^4^. Therefore, when the pH value is in the range from 6 to 10, both adsorption and coprecipitation contribute to the significant increase of the removal efficiency of Cu^2+^, where coprecipitation plays a leading role. For this reason, a pH of 6 should be used as the best experimental condition in future studies. Figure 4 also shows that adsorption capacities of TiO_2_@GAC were higher than those of AC for dyes and Cu^2+^. Previous work showed that mesoporous TiO_2_ was an excellent adsorbent for dyes and heavy metal, with higher adsorption capacities for dyes and heavy metal^7^ than those of GAC or TiO_2_@GAC. Nevertheless, mesoporous TiO_2_ is difficult to be separated from aqueous solution for recovery and reuse. The combination of GAC and TiO_2_ can make up for the shortcomings of TiO_2_ and thereby allowing wide application in wastewater treatment^12^.

### Effect of time on adsorption

**Figure 5:**
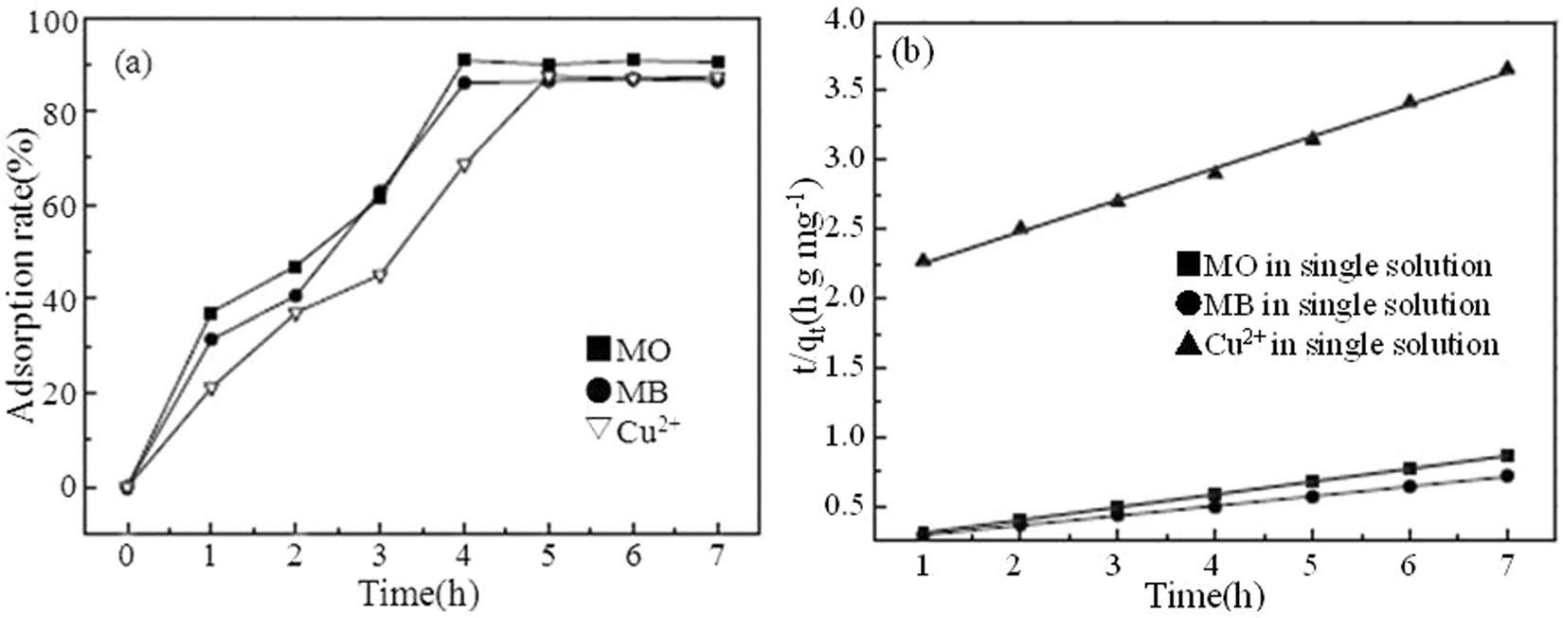
Effect of time on adsorption of MO, MB and Cu^2+^ on TiO_2_@GAC (**a**) and Fit of kinetic data to pseudo-second-order model in single component systems (**b**).

Figure 5 depicts the effect of time on adsorption of MO (MB) and Cu^2+^ on TiO_2_@GAC in single systems. The time for MO and MB to reach the adsorption equilibrium is 4 h, and the time for Cu^2+^ to reach the adsorption equilibrium is 5 h. At the initial stage, the adsorption rates of all the three substances increase rapidly, which may be due to the fact that there are abundant adsorption sites on TiO_2_@GAC for the adsorption of dyes and Cu^2+^. With the lapse of time, more dye molecules and Cu^2+^ are adsorbed on the surface of TiO_2_@GAC, resulting in less available sites. Meanwhile, the concentration of dyes and Cu^2+^ in the solution also decreases. Therefore, the adsorption effect is reduced.

**Figure 6:**
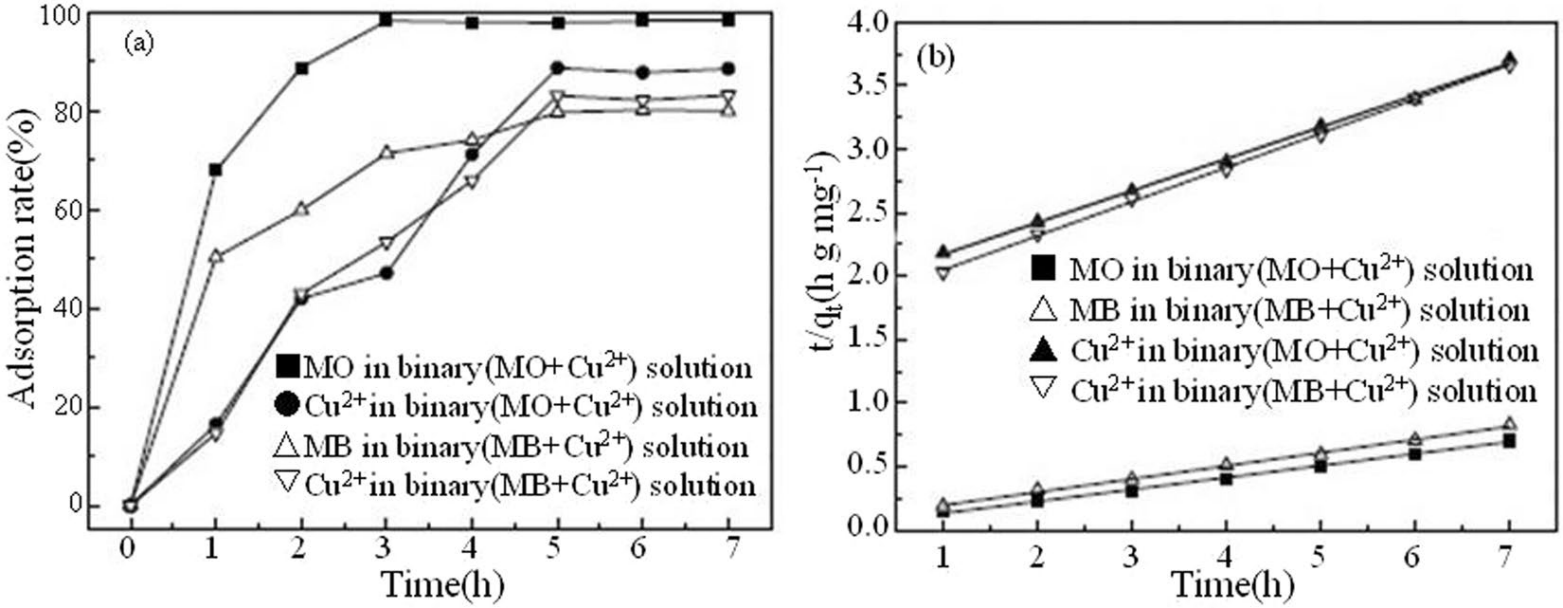
Effect of time on the adsorption of MO, MB and Cu^2+^ on TiO_2_@GAC (**a**) and Fit of kinetic data to pseudo-second-order model in binary component systems (**b**).

Figure 6 depicts the effect of time on adsorption of MO (MB) and Cu^2+^ on TiO_2_@GAC in binary systems. In the binary system with MO and Cu^2+^, the adsorption equilibrium times of MO and Cu^2+^ are 3 h and 5 h, respectively. Both substances show a higher adsorption rate compared with single systems. Furthermore, the increase of the adsorption rates during the initial stage is also significantly faster compared with single systems. This may be due to the synergistic effect between the positively charged Cu^2+^ and the negative charged MO. In the binary system with MB and Cu^2+^, the adsorption equilibrium times of MB and Cu^2+^ are both 5 h. Both substances show a lower adsorption rate compared with single systems. This may be due to the competition between the positively charged Cu^2+^ and the positively charged MB.

The pseudo-first-order model and pseudo-second-order model are built to describe the adsorption kinetics. The equations of the two models are as follows^20^,

Pseudo-first-order model,

2 $$\mathrm{ln}({{\rm{q}}}_{{\rm{e}}}-{{\rm{q}}}_{{\rm{t}}})=\,\mathrm{ln}\,{{\rm{q}}}_{{\rm{e}}}-{{\rm{K}}}_{1}{\rm{t}}$$

Pseudo-second-order model,

3 $$\frac{{\rm{t}}}{{{\rm{q}}}_{{\rm{t}}}}=\frac{1}{{{\rm{K}}}_{2}{{\rm{q}}}_{{\rm{m}}}^{2}}+\frac{{\rm{t}}}{{{\rm{q}}}_{{\rm{m}}}}$$

where q_m_ is the adsorption capacity (mg/g) at the equilibrium, q_t_ is the adsorption capacity (mg/g) at time t, K_1_ is the adsorption equilibrium rate constant (h^−1^) of the pseudo-first-order model, and K_2_ is the adsorption equilibrium rate constant (g/(mg·h)) of the pseudo-second-order model.

Table 1 lists the kinetic model parameters for the adsorption of MO, MB and Cu^2+^ on TiO_2_@GAC in single systems and binary systems. According to the correlation (R^2^), MO, MB and Cu^2+^ in single systems and binary systems all comply with the pseudo-second-order kinetic model (Figs 5(b) and 6(b)). This indicates that the adsorption of MO, MB and Cu^2+^ on TiO_2_@GAC is a chemical adsorption process^21^.

**Table 1:** Pseudo-first-order, pseudo-second-order kinetics constants for MO, MB and Cu^2+^ adsorption in single and binary systems.

System	Adsorbate	Pseudo-first			Pseudo-second		
		q_m,cal_ (mg/g)	K_1_ (h^−1^)	R^2^	q_m,cal_ (mg/g)	K_2_ (g/(mg · h))	R^2^
Single	MO	27.08	1.3715	0.7852	10.6	3.99 × 10^−2^	0.9897
	MB	31.48	1.1761	0.8702	14.47	2.05 × 10^−2^	0.9796
	Cu^2+^	13.38	1.2565	0.8331	4.56	2.36 × 10^−2^	0.98
Binary	MO (MO + Cu^2+^)^a^	5.75	1.2126	0.8651	10.93	2.56 × 10^−1^	0.9972
	Cu^2+^ (MO + Cu^2+^)^b^	12.36	1.2044	0.8569	3.85	3.68 × 10^−2^	0.9756
	MB (MB + Cu^2+^)^a^	12.64	0.9591	0.9316	9.66	1.17 × 10^−1^	0.9974
	Cu^2+^ (MB + Cu^2+^)^b^	7.51	1.1264	0.6767	3.87	3.64 × 10^−2^	0.9693

^a^Concentration of MO or MB was fixed, while changing concentration of Cu^2+^.

^b^Concentration of Cu^2+^ was fixed, while changing concentration of MO or MB.

### Effect of concentration on adsorption in single systems

**Figure 7:**
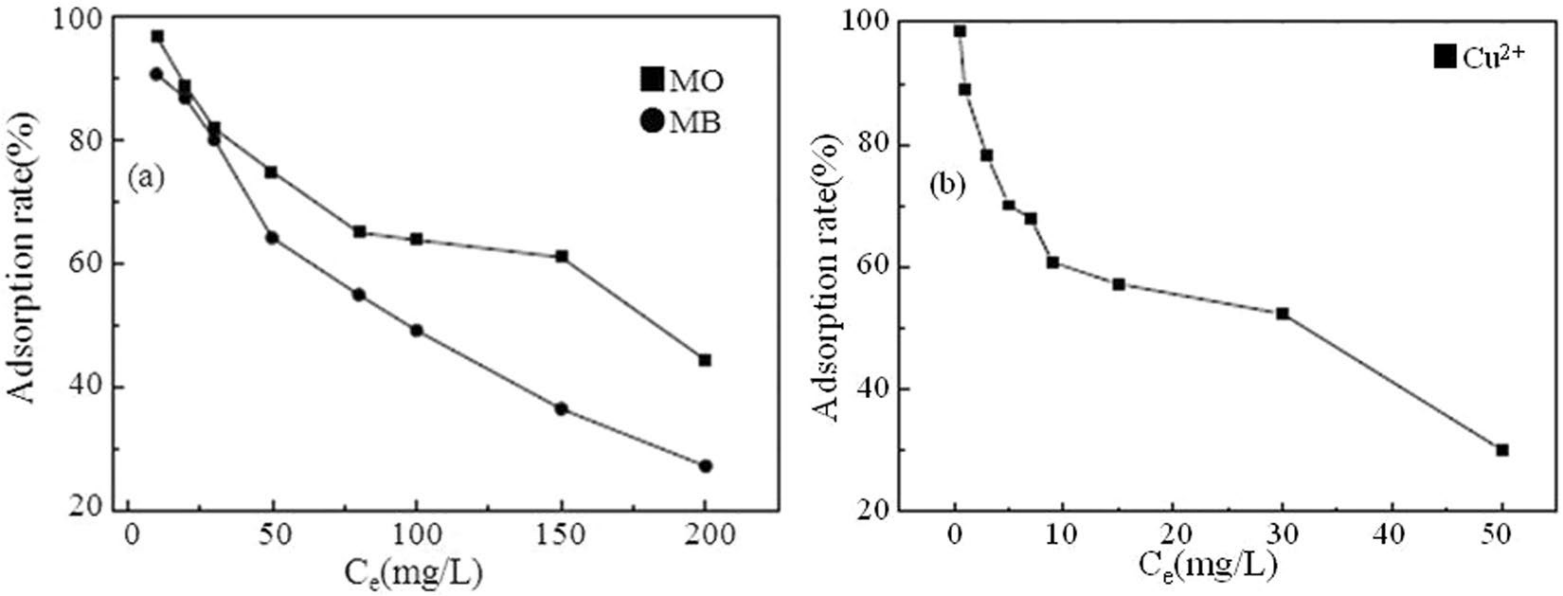
Effect of concentration on the adsorption of MO, MB (**a**) and Cu^2+^ (**b**) on TiO_2_@GAC in single component systems.

Figure 7 depicts the effect of concentration on adsorption of dyes and Cu^2+^ on TiO_2_@GAC in single systems. It can be seen from Fig. 7 that, the adsorption rates of both dyes and Cu^2+^ on TiO_2_@GAC decrease with the increase of the initial concentration. When the adsorption time and the concentration of adsorbent are constant, the adsorption sites on the adsorbent surface decrease with the increase of the concentration of MO (MB) and Cu^2+^, thus reducing the adsorption rates.

The Langmuir and Freundlich isotherm adsorption equations are used to process the experimental data. The linear equations of Langmuir^22^ (Eq. 4) and Freundlich^23^ (Eq. 5) isothermal models are as follows,

4 $$\frac{{{\rm{C}}}_{{\rm{e}}}}{{{\rm{q}}}_{{\rm{e}}}}=\frac{{{\rm{C}}}_{{\rm{e}}}}{{{\rm{q}}}_{{\rm{m}}}}+\frac{1}{{{\rm{q}}}_{{\rm{m}}}{{\rm{K}}}_{{\rm{L}}}}$$

5 $$\mathrm{ln}\,{{\rm{q}}}_{{\rm{e}}}=\,\mathrm{ln}\,{{\rm{K}}}_{{\rm{F}}}+\frac{1}{{\rm{n}}}\,\mathrm{ln}\,{{\rm{C}}}_{{\rm{e}}}$$

where q_e_ is the equilibrium adsorption capacity per unit mass of TiO_2_@GAC for dyes and Cu^2+^, C_e_ is the equilibrium concentration, K_L_ is the Langmuir equilibrium adsorption constant, q_m_ is the maximum adsorption capacity per unit mass of TiO_2_@GAC, K_F_ is the capacity coefficient and n is the intensity factor.

According to R^2^ in Table 2, the isothermal adsorption models of MO, MB and Cu^2+^ in single systems all comply with the Langmuir model, which indicates that the adsorption process of MO, MB and Cu^2+^ on TiO_2_@GAC is monomolecular adsorption^24^. The maximum adsorption capacities of MO, MB and Cu^2+^ are 32.36 mg/g, 25.32 mg/g and 23.42 mg.

**Table 2:** Adsorption isotherm constants for MO, MB and Cu^2+^ adsorption in single component systems.

Adsorbate	q_m_ (mg/g)	Langmuir model		Freundlich model		
		K_L_	R^2^	K_F_	n	R^2^
MO	32.36	1.18 × 10^−2^	0.9939	0.5	1.29	0.9323
MB	25.32	1.53 × 10^−2^	0.9849	0.91	1.64	0.9537
Cu^2+^	23.42	3.68 × 10^−2^	0.9932	0.94	1.27	0.9871

### Effect of concentration on adsorption in binary systems

**Figure 8:**
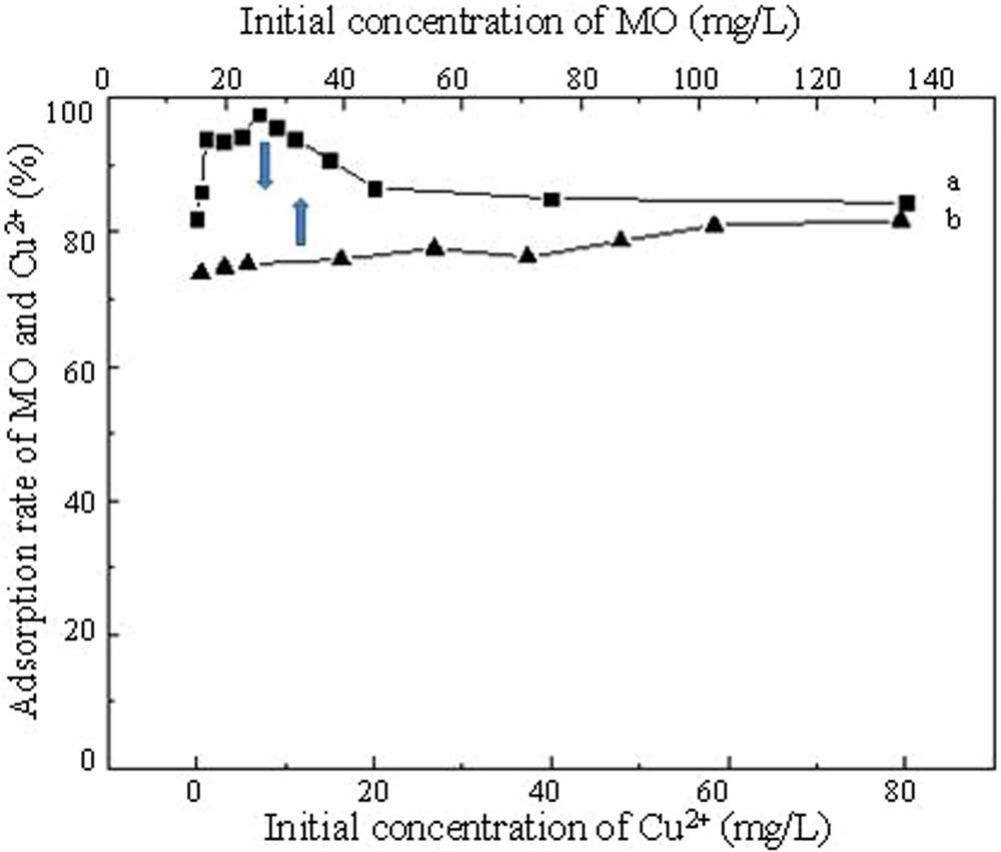
Effect of initial concentration Cu^2+^ on adsorption MO (**a**) and effect of initial concentration of MO on adsorption Cu^2+^ (**b**).

It can be seen from Fig. 8(a) that, in the binary system with a constant concentration of MO, the adsorption rate of MO first increased and then decreased with concentration of Cu^2+^. In addition to the adsorption of TiO_2_@GAC itself, the increase in the adsorption rate of MO may also be due to the synergistic effect between positively charged Cu^2+^ and negatively charged MO. However, as the concentration of Cu^2+^ increased, the adsorbed MO may be replaced by Cu^2+^, causing decrease in removal rate of MO. Although the removal rate of MO decreased, the adsorption rate was still above 85%. However, in the presence of MO, with a constant concentration of Cu^2+^, the adsorption rate of Cu^2+^ TiO_2_@GAC remained almost unchanged with concentration of MO, as shown in Fig. 8(b). This was probably because Cu^2+^ entered the pores in activated carbon and got adsorbed earlier than organic substance MO with a higher molecular weight. Therefore, the effect of MO on the adsorption of Cu^2+^ was relatively small and almost negligible.

**Figure 9:**
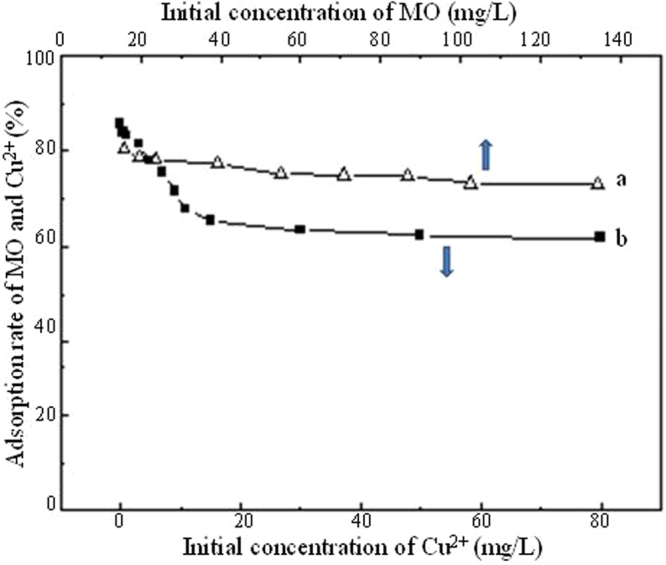
Effect of initial concentration Cu^2+^ on adsorption MB (**a**) and effect of initial concentration of MB on adsorption Cu^2+^ (**b**).

It can be seen from Fig. 9 that, in the binary system consisting of MB and Cu^2+^, in the presence of Cu^2+^, with a constant concentration of MB, the adsorption rate of MB decreases with the increase of the concentration of Cu^2+^. This is because there is a competitive relationship between positively charged Cu^2+^ and positively charged MB. As the concentration of Cu^2+^ increases, the removal rate of MB decreases. In the presence of MB, the adsorption rate of Cu^2+^ also remains almost unchanged as the concentration increases. This is also probably because Cu^2+^ get adsorbed earlier than the organic matter MB with a higher molecular weight.

## Conclusions

Granular activated carbon-supported titanium dioxide particles were prepared with the sol-gel method and their adsorption performance for dyes and Cu^2+^ were studied. In both single systems and binary systems, the adsorption behaviors of MO, MB and Cu^2+^ by TiO_2_@GAC all complies with the pseudo-second-order kinetic model. In single systems, the adsorption isotherms of both dyes and Cu^2+^ on TiO_2_@GAC comply with the Langmuir model, which indicates that the adsorption process of TiO_2_@GAC is monomolecular chemisorption. The pH has significant effect on the adsorption of dyes and Cu^2+^. For Cu^2+^, coprecipitation also contributes and plays a leading role. In the binary system consisting of MO and Cu^2+^, the adsorption rate of MO increases first and then decrease while the adsorption rate of Cu^2+^ remains almost unaffected.

The increase in the adsorption rate of MO is due to the adsorption and the synergistic effect between Cu^2+^ and MO, while the unaffected adsorption rate of Cu^2+^ and the subsequent decrease in the adsorption rate of MO may be due to the preferential adsorption of Cu^2+^ on TiO_2_@GAC. Similarly, in the binary system consisting of MB and Cu^2+^, the adsorption rate of MB increases first and then decrease while the adsorption rate of Cu^2+^ remains almost unaffected. The increase in the adsorption rate of MB is due to the adsorption of TiO_2_@GAC, while the subsequent decrease in the adsorption rate of MB is due to the competition between Cu^2+^ and MB. The above experimental results provide a certain theoretical basis for the removal of dyes and heavy metal ions with TiO_2_@GAC in practical applications.
